# Enhancing the efficiency of the *Pichia pastoris AOX1* promoter via the synthetic positive feedback circuit of transcription factor Mxr1

**DOI:** 10.1186/s12896-018-0492-4

**Published:** 2018-12-27

**Authors:** Ching-Hsiang Chang, Hao-An Hsiung, Kai-Lin Hong, Ching-Tsan Huang

**Affiliations:** Department of Biochemical Science and Technology, National Taiwan University, No. 1, Sec. 4, Roosevelt Road, Taipei, 10617 Taiwan

**Keywords:** *Pichia pastoris*, Recombinant protein expression, *AOX1* promoter, Methanol, Methanol expression regulator 1, Synthetic gene circuit, Transcriptional reprogramming

## Abstract

**Background:**

The methanol-regulated *AOX1* promoter (P_*AOX1*_) is the most widely used promoter in the production of recombinant proteins in the methylotrophic yeast *Pichia pastoris*. However, as the tight regulation and methanol dependence of P_*AOX1*_ restricts its application, it is necessary to develop a flexible induction system to avoid the problems of methanol without losing the advantages of P_*AOX1*_. The availability of synthetic biology tools enables researchers to reprogram the cellular behaviour of *P. pastoris* to achieve this goal*.*

**Results:**

The characteristics of P_*AOX1*_ are highly related to the expression profile of methanol expression regulator 1 (Mxr1). In this study, we applied a biologically inspired strategy to reprogram regulatory networks in *P. pastori*s. A reprogrammed *P. pastoris* was constructed by inserting a synthetic positive feedback circuit of Mxr1 driven by a weak *AOX2* promoter (P_*AOX2*_). This novel approach enhanced P_*AOX1*_ efficiency by providing extra Mxr1 and generated switchable Mxr1 expression to allow P_*AOX1*_ to be induced under glycerol starvation or carbon-free conditions. Additionally, the inhibitory effect of glycerol on P_*AOX1*_ was retained because the synthetic circuit was not activated in response to glycerol. Using green fluorescent protein as a demonstration, this reprogrammed *P. pastoris* strain displayed stronger fluorescence intensity than non-reprogrammed cells under both methanol induction and glycerol starvation. Moreover, with single-chain variable fragment (scFv) as the model protein, increases in extracellular scFv productivity of 98 and 269% were observed in Mxr1-reprogrammed cells under methanol induction and glycerol starvation, respectively, compared to productivity in non-reprogrammed cells under methanol induction.

**Conclusions:**

We successfully demonstrate that the synthetic positive feedback circuit of Mxr1 enhances recombinant protein production efficiency in *P. pastoris* and create a methanol-free induction system to eliminate the potential risks of methanol.

**Electronic supplementary material:**

The online version of this article (10.1186/s12896-018-0492-4) contains supplementary material, which is available to authorized users.

## Background

The methylotrophic yeast *Pichia pastoris* has been extensively used in the production of recombinant proteins because it provides the advantages of post-translational modification in a eukaryotic single-cell system. Protein production in *P. pastoris* is typically driven by the *AOX1* promoter (P_*AOX1*_), which occurs in response to methanol induction due to its strong and regulatable characteristics [1, 2]. To date, more than 5000 recombinant proteins have been successfully produced in *P. pastoris* [3, 4]. Despite the potential of the *P. pastoris* expression system, tightly regulated P_*AOX1*_ limits expression to restrictive conditions, with the presence of repressing carbon sources significantly decreasing recombinant protein expression during the methanol-induction phase [5]. Residual carbon sources can be removed by medium replacement prior to methanol induction, though this process is not applicable in large-scale production [6]. In addition, methanol is a toxic and flammable compound that presents some potential problems as the inducer or carbon source [7–11].

The development of synthetic biology tools has enabled researchers to reprogram cellular behaviour in *P. pastoris* to avoid the drawbacks of tight P_*AOX1*_ regulation. The use of alternative promoters or depressed P_*AOX1*_ variants both provided solutions for methanol-independent production [12–14]. The improvement of induction efficiency of P_*AOX1*_ under non-methanol carbon sources was achieved by reprogramming the carbon metabolic pathway of *P. pastoris* [15, 16]. However, such a strategy might interrupt carbon metabolism and result in growth defects. An alternative approach is to reprogram transcriptional regulation of P_*AOX1*_. Although the regulation mechanism of P_*AOX1*_ is not fully understood, several transcription factors involved in P_*AOX1*_ regulation have been identified. In response to different carbon sources, P_*AOX1*_ has three regulated stages of gene expression including repression, derepression, and activation [17]. Among carbon regulation of P_*AOX1*_, the transcriptional activator Mxr1 is constitutively expressed and plays a crucial role in P_*AOX1*_ derepression and activation processes [18–20]. The Nrg1 repressor participates in the inhibition mechanism by competing for Mxr1 binding elements in P_*AOX1*_ [21]; Nrg1 can be down-regulated by switching the carbon source from glycerol to methanol [22], though the detailed expression pattern remains unclear. During the activation process, the activators Prm1 and Mit1 are up-regulated by methanol to activate P_*AOX1*_ expression [19, 20]. In previous studies, the P_*AOX1*_-based methanol free expression system could be achieved by deletion of three repressors (Nrg1, Mig1 and Mig2) and overexpression of one activator (Mit1) [3], or by derepressed overexpression of Mxr1 or Mit1 [23]. However, *NRG1* deletion might lead to the potential risk of growth defects [21]. Hence, developing the synthetic circuits to provide an efficient approach for controlling the characteristics of P_*AOX1*_, remains a challenge.

In this study, we reprogrammed regulatory networks in *P. pastoris* through biological inspiration of another methylotrophic yeast: *Hansenula polymorpha*. Unlike P_*AOX1*_ in *P. pastori*s, glycerol does not interfere with the methanol-induced efficiency of P_*MOX*_, the alcohol oxidase promoter in *H. polymorpha*. In addition to induction by methanol, P_*MOX*_ can express recombinant genes via a carbon starvation strategy [17, 24]. The regulatory difference between P_*AOX1*_ and P_*MOX*_ results from upstream transcriptional networks in cells rather than due to promoter sequences alone [25]. Interestingly, this phenomenon might be related to the different expression pattern between *P. pastoris* Mxr1 and its orthologous gene (HPODL00650) in *H. polymorpha*, as HPODL00650 is up-regulated by methanol [26]. We speculate the existence of positive feedback regulation of HPODL00650 in *H. polymorpha,* which might contribute to the flexible activation of P_*MOX1*_*.* Therefore, to mimic the expression pattern of HPODL00650, a reprogrammed *P. pastoris* strain was constructed by inserting a synthetic positive autoregulation circuit of Mxr1. In addition to endogenous Mxr1, exogenous Mxr1 was driven by the methanol-regulated *AOX2* promoter (P_*AOX2*_), a promoter that is weaker than P_*AOX1*_. This novel strategy did not affect cells in the repression condition, thus maintaining tight regulation of P_*AOX1*_ and preventing growth defects. We demonstrate herein that the transcriptional efficiency of P_*AOX1*_ was enhanced and that the interference due to residual repressing carbon sources was reduced. These Mxr1-reprogrammed cells show great potential for broader applications.

## Results

### Mxr1-reprogrammed cells had altered GFP expression but did not show growth defects

**Fig. 1:**
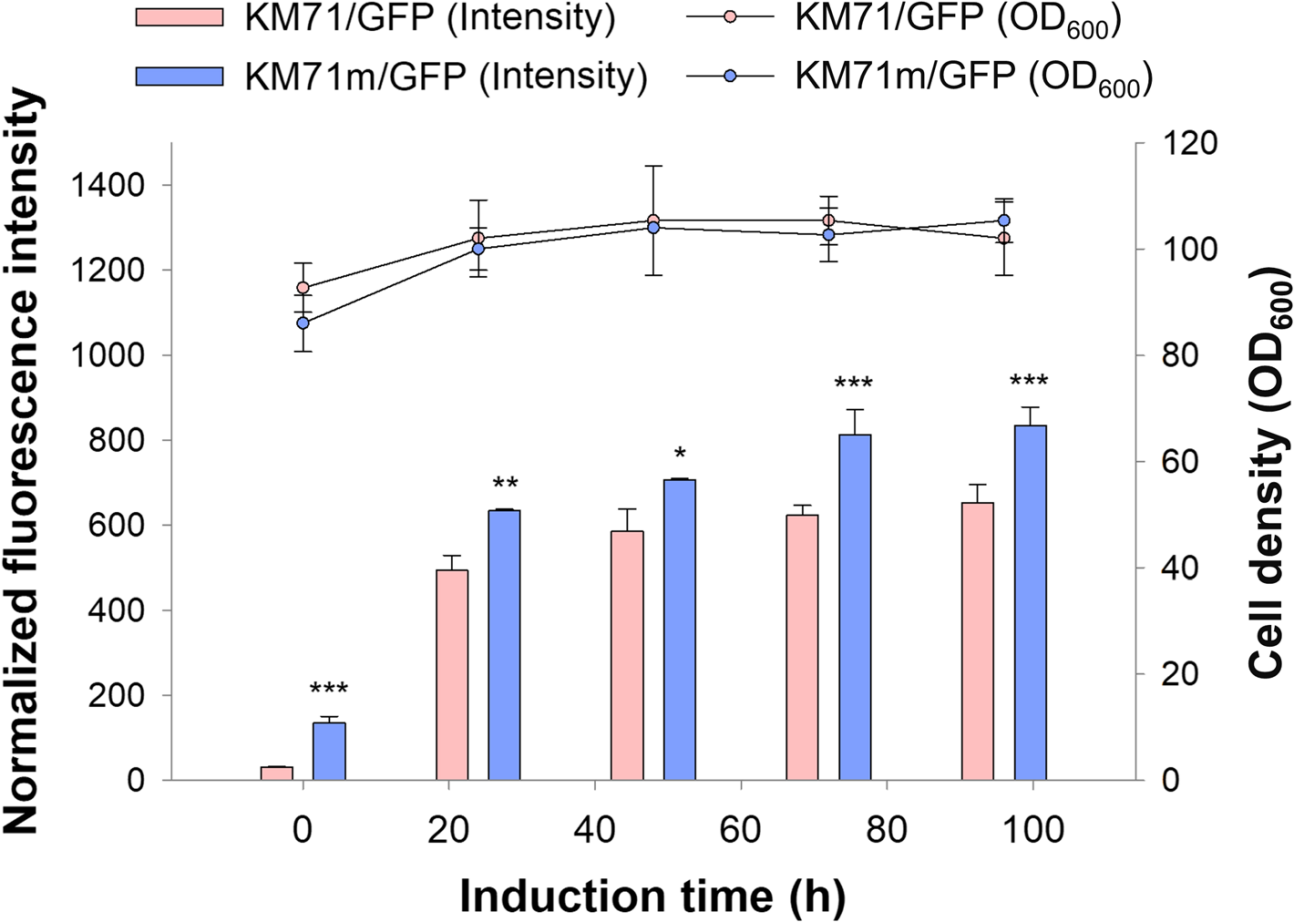
Mxr1-reprogrammed cells had altered GFP expression but did not show growth defects. After being cultured in BMGY (1% glycerol), cells were cultured in BMMY and induced with 0.5% methanol daily. Cell density (OD_600_) is presented by a line and scatter plot, and the normalized fluorescence intensity is presented by a bar plot. Error bars represent the standard deviation of three biological replicates. The independent-sample t-test was used to determine significance. *, *p* < 0.05; **, *p* < 0.01; ***, *p* < 0.005

Mxr1-reprogrammed cells were constructed by exogenous expression of Mxr1 controlled by the P_*AOX2*_ in a clone carrying nine copies of the P_*AOX1*_-regulated GFP expression cassette. Non-reprogrammed *P. pastoris* KM71/GFP and reprogrammed *P. pastoris* KM71m/GFP were compared to evaluate the effects of the synthetic Mxr1 circuit on cell growth and heterologous gene expression. Figure 1 illustrates that *P. pastoris* KM71m/GFP cells exhibited stronger fluorescence intensity than did *P. pastoris* KM71/GFP cells during methanol induction. In addition, no significant differences in cell growth were found throughout the experiments, suggesting that expression of Mxr1 by the mild P_*AOX2*_ promoter did not cause growth defects. However, as evidenced by GFP production prior to methanol induction, an altered Mxr1 expression pattern might result in the potential risk of P_*AOX1*_ leakage.

### P_*AOX1*_ remained controllable in Mxr1-reprogrammed cells

**Fig. 2:**
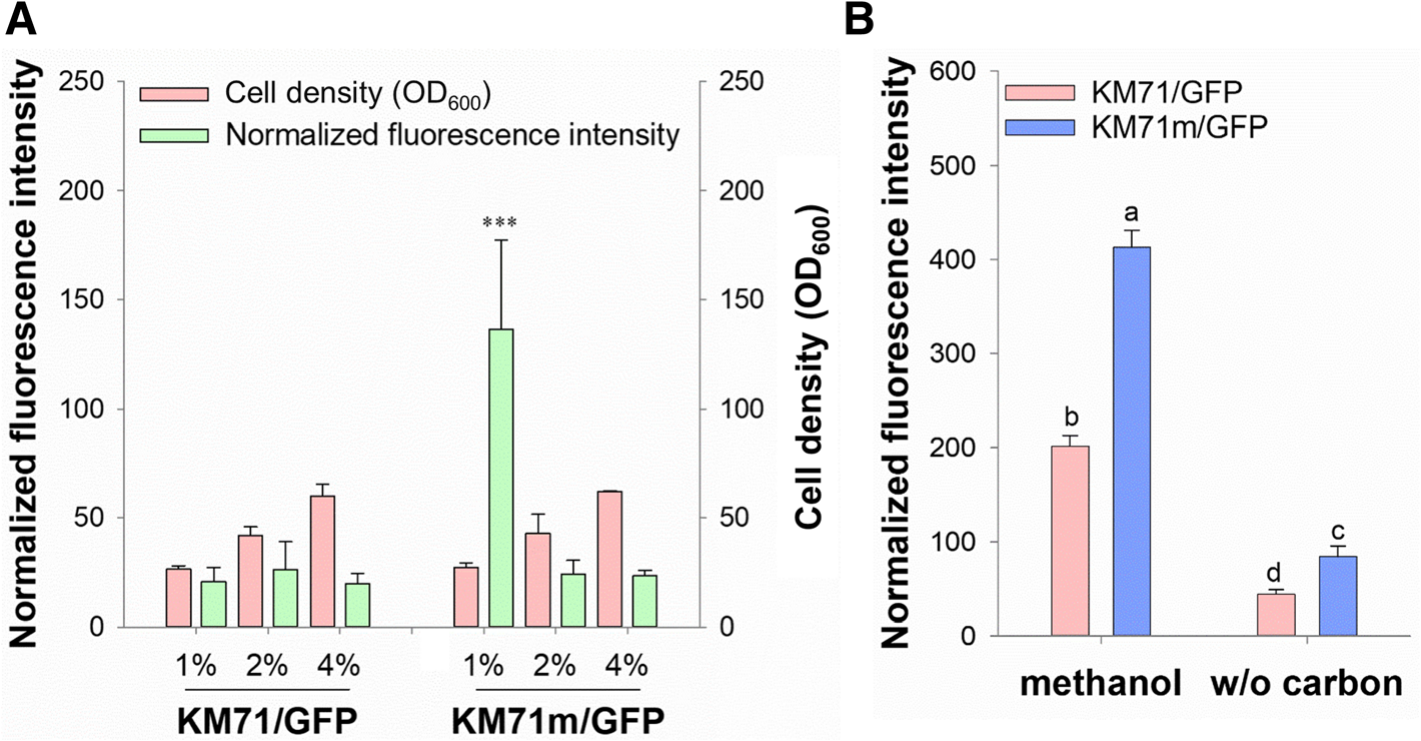
P_*AOX1*_ remained controllable in Mxr1-reprogrammed cells. **a** Detection of the cell density (OD_600_) and normalized fluorescence intensity of cells grown in different concentrations of glycerol for 24 h. The error bars represent the standard deviation of three biological replicates. Two-way analysis of variance (ANOVA) and the Tukey test were used to determine significance. ***, *p* < 0.005. **b** After being cultured in BMGY (2% glycerol), the cells were cultured in BMNY or BMMY. Detection of the normalized fluorescence intensity of cells grown in different carbon sources for 3 h. The error bars represent the standard deviation of three biological replicates. Two-way ANOVA and the Tukey test were used to determine significance. Groups with different letters are significantly different

Several studies have suggested that P_*AOX1*_ was leaky under derepression conditions, especially in bioreactor cultivations [27–29]. Therefore, to assess the level of P_*AOX1*_ leakage, cells were cultured in different concentrations of glycerol for 24 h. As shown in Fig. 2a, the cell density (OD_600_) of both *P. pastoris* KM71/GFP and *P. pastoris* KM71m/GFP cell cultures increased with a rise in glycerol concentration, indicating that the carbon source was the limiting factor in a 24-h culture; furthermore, no growth defects of the reprogrammed cells were found. As expected, only the baseline fluorescence intensity was observed for *P. pastoris* KM71/GFP cells under 1, 2 and 4% glycerol. In contrast, the fluorescence intensity of *P. pastoris* KM71m/GFP cells significantly increased under conditions of 1% glycerol, though no significant difference in *P. pastoris* KM71/GFP cells under 2 or 4% glycerol was found. Based on these results, it was plausible that P_*AOX1*_ in *P. pastoris* KM71m/GFP cells was activated by glycerol depletion. To verify this assumption, a glycerol-depletion condition was established by replacing 2% glycerol with a carbon-free medium. As shown in Fig. 2b, *P. pastoris* KM71m/GFP cells displayed enhanced GFP expression, regardless of whether they were induced by methanol. Compared with *P. pastoris* KM71/GFP under methanol induction, the relative GFP production by *P. pastoris* KM71m/GFP cells was 205 ± 9 and 42 ± 6% under methanol induction and carbon starvation, respectively. These results suggested that reprograming Mxr1 improved protein production efficiency and resulted in flexible activation conditions without interfering with the controllable characteristics of P_*AOX1*._

### Positive feedback of Mxr1 increased the transcriptional efficiency of P_*AOX1*_ and broke the Mxr1 titration effect

**Fig. 3:**
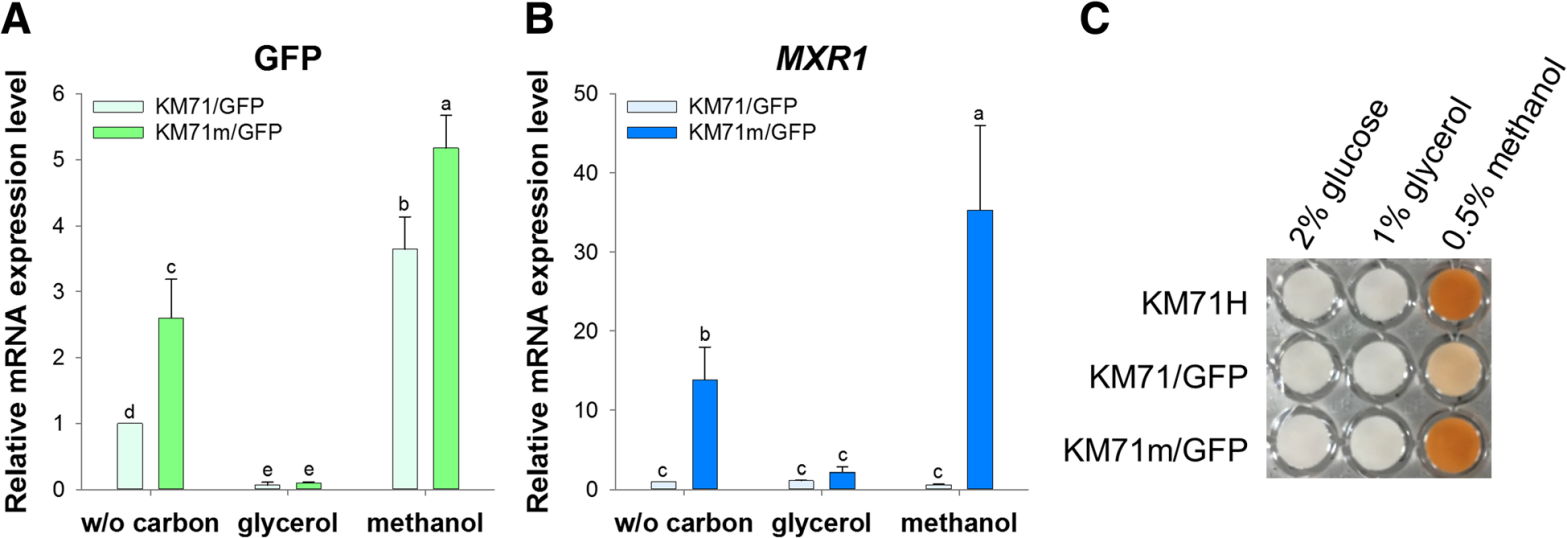
Positive feedback of Mxr1 increased the transcriptional efficiency of P_*AOX1*_ and broke the Mxr1 titration effect. mRNA was extracted from cells cultured in different carbon sources for 3 h. **a** GFP. **b** *MXR1*. mRNA levels in each sample were normalized to 18S rRNA. The relative expression level for each gene was normalized to the control grown under the carbon-free condition. The error bars represent the standard deviation of three biological replicates. Two-way ANOVA and the Tukey test were used to determine significance. Groups with different letters are significantly different. **c** AOX activity in different cells in response to different carbon sources

To further confirm that the increase in GFP expression resulted from enhanced transcriptional efficiency, mRNA expression levels of GFP and Mxr1 were determined by quantitative reverse transcription-polymerase chain reaction (RT-qPCR). In the presence of glycerol, mRNA expression levels of GFP in both reprogrammed and non-reprogrammed cells were repressed, with no significant difference. In contrast, the transcriptional efficiency of P_*AOX1*_ in *P. pastoris* KM71m/GFP cells was significantly increased in response to methanol induction or carbon starvation (Fig. 3a). As shown in Fig. 3b, Mxr1 was, not surprisingly, constitutively expressed in *P. pastoris* KM71/GFP, and equal Mxr1 expression was detected in *P. pastoris* KM71m/GFP cells in response to glycerol. However, the expression levels of Mxr1 showed 13.9-fold and 35.4-fold increases in *P. pastoris* KM71m/GFP cells under carbon starvation and methanol induction, respectively. Although Mxr1 expression showed higher fold increases compared to GFP after reprogramming, the transcript levels of Mxr1 were lower than those of GFP due to the weak P_*AOX2*_(Additional file 1: Figure S1). These results suggested that the positive feedback circuit of Mxr1 enhanced P_*AOX1*_ transcriptional efficiency without breaking the inhibitory effect of glycerol.

According to the study of Camara et al., the Mxr1 titration effect is a plausible explanation for the observed transcriptional attenuation of methanol-induced genes in increasingly used P_*AOX1*_-regulated expression cassettes [30]. To verify whether this Mxr1 titration effect can be disrupted by exogenous expression of Mxr1, the methanol utilization capacities of *P. pastoris* KM71H, *P. pastoris* KM71/GFP, and *P. pastoris* KM71m/GFP cells were determined (Fig. 3c). As evidenced by a colorimetric assay, AOX activity in *P. pastoris* KM71/GFP cells was weaker than that in *P. pastoris* KM71H cells in response to methanol. However, the defect in methanol utilization was recovered in *P. pastoris* KM71m/GFP cells, suggesting that the expression level of Mxr1 was a bottleneck for heterologous gene expression. Hence, breaking the Mxr1 titration effect by a synthetic Mxr1 circuit is expected to increase the potential of using a high copy-number strategy.

### Mxr1-reprogrammed cells were inducible under non-restricted conditions

**Fig. 4:**
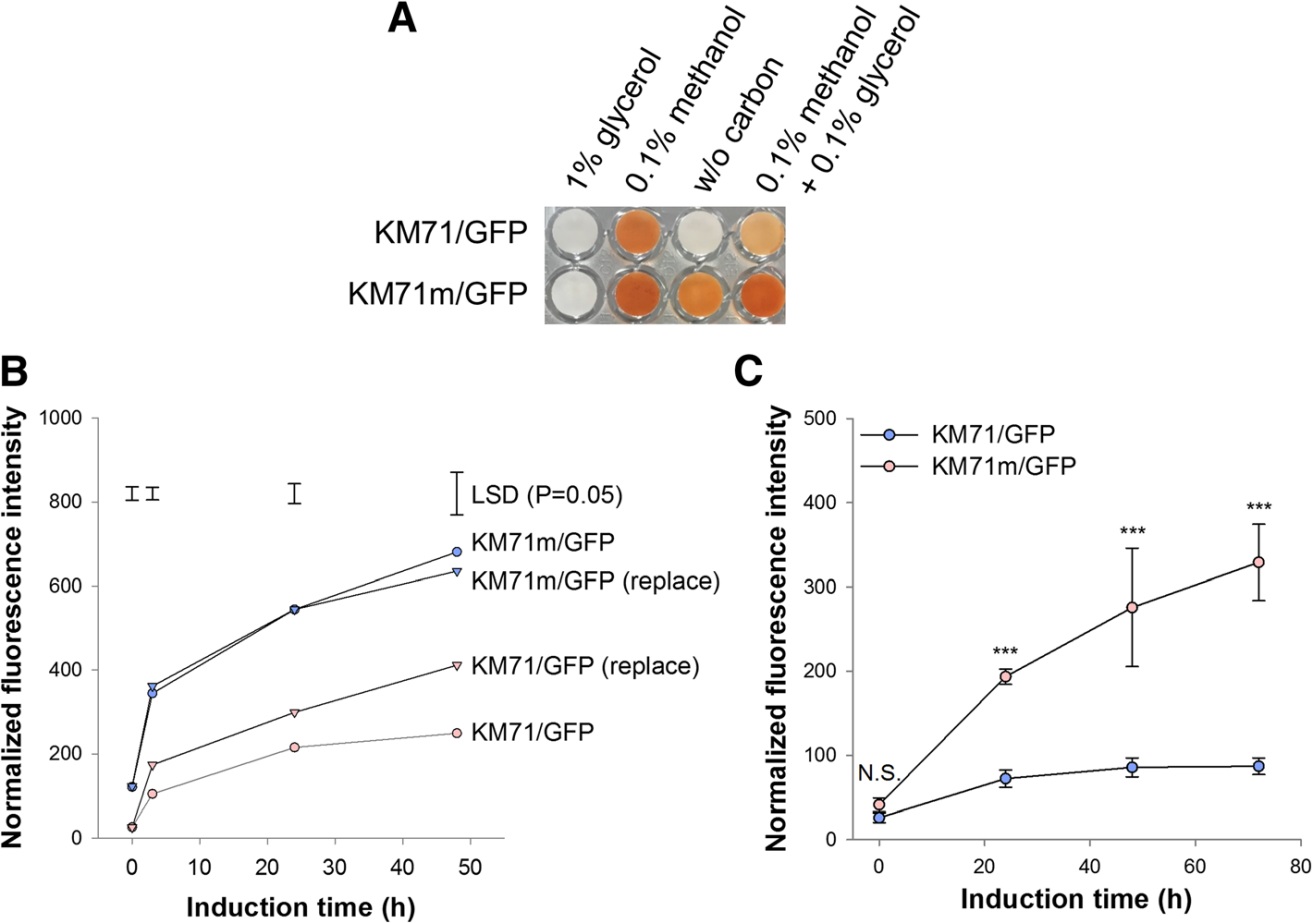
Mxr1-reprogrammed cells were induced under broad conditions. **a** AOX activity in different cells in response to different carbon sources. **b** After being cultured in BMGY (2% glycerol), cells were concentrated in the original medium, or the medium was replaced by fresh medium without residual glycerol. The cells were then induced with 0.5% methanol per day. The results represent the mean of three independent biological replicates. Two-way ANOVA and the least significant difference (LSD) test were used to determine significance (*p* = 0.05). **c** After being cultured in BMGY (2% glycerol), cells were cultured in BMGY and induced with 0.33% glycerol per day. The error bars represent the standard deviation of three biological replicates. The independent-sample t-test was used to determine significance. NS, *p* > 0.05; ***, *p* < 0.005

Figure 4a presents the AOX activity of *P. pastoris* KM71/GFP and *P. pastoris* KM71m/GFP cells in response to different carbon combinations. The tight P_*AOX1*_ regulation of *P. pastoris* KM71/GFP cells resulted in no AOX activity under glycerol and carbon-free conditions, and methanol-induction efficiency was also interfered by low concentration of glycerol. Conversely, *P. pastoris* KM71m/GFP cells not only bypassed the interference caused by small amount of glycerol but also were induced under the carbon-free condition. These results suggested that the flexible characteristic of P_*MOX*_ was achieved by reprogramming the regulation pattern of Mxr1.

Although the fluorescence intensity of *P. pastoris* KM71/GFP was enhanced by medium replacement (Fig. 4b), the intensity of *P. pastoris* KM71m/GFP with or without medium replacement was significantly higher than that of *P. pastoris* KM71/GFP with medium replacement. These results suggested that Mxr1 reprogramming overcame the interference of residual repressive carbon and resulted in a smooth transition between glycerol and methanol. In addition to the above advantage, Mxr1 reprogramming showed great potential in the development of a methanol-free induction system. As shown in Fig. 4c, the fluorescence intensity of *P. pastoris* KM71m/GFP increased significantly by the daily addition of 0.33% glycerol, whereas only baseline intensity was detected for *P. pastoris* KM71/GFP. These results indicated that Mxr1-reprogrammed *P. pastoris* is inducible under glycerol depletion condition.

### Application of Mxr1-reprogrammed cells in the production of scFv

**Fig. 5:**
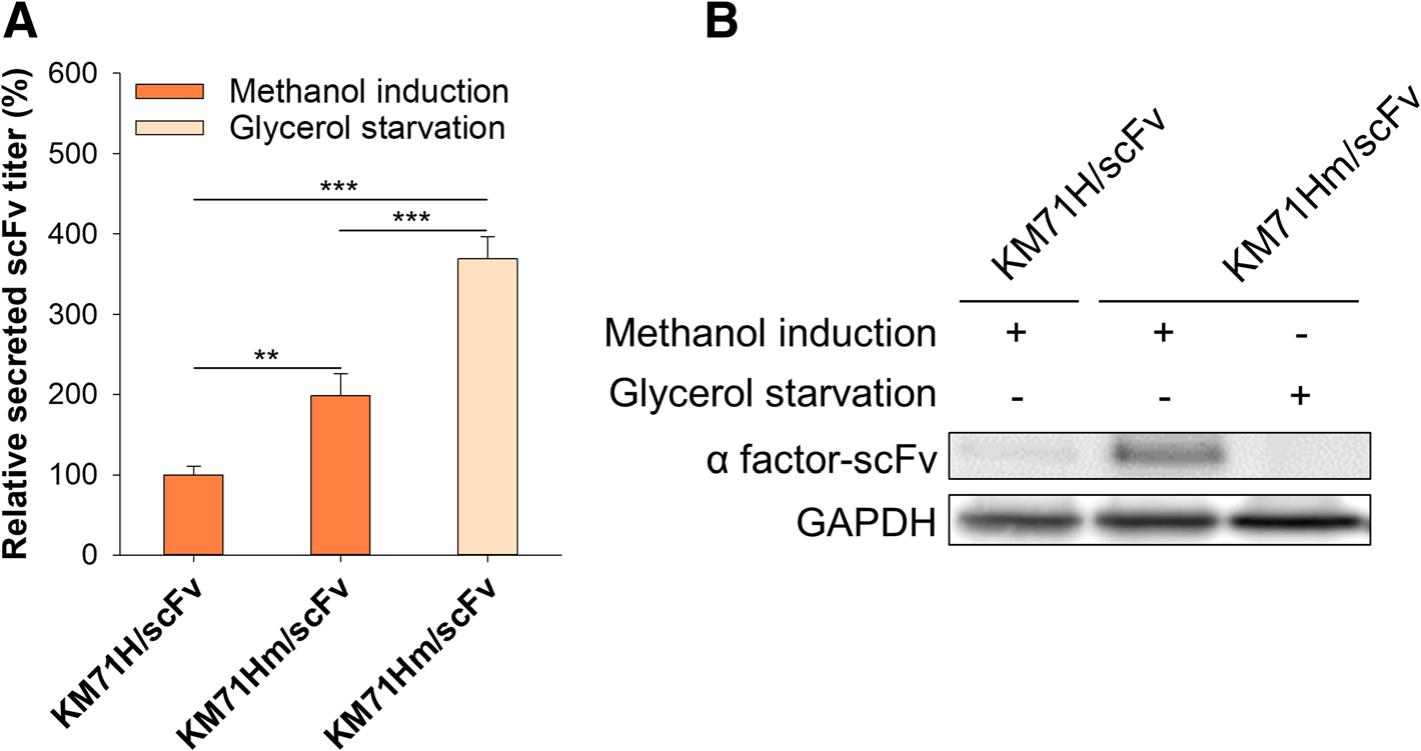
Synthetic circuit of Mxr1 enhanced scFv expression. After being cultured in BMGY (1% glycerol), cells were cultured in BMMY and induced with 1% methanol daily. **a** The relative secreted scFv titre is presented. The error bars represent the standard deviation of three biological replicates. One-way ANOVA and the Tukey test were used to determine significance. *, *p* < 0.05; ***, *p* < 0.005. **b** Intracellular proteins after induction were examined by western blotting using an anti-his antibody. GAPDH was used as the loading control

To demonstrate the application feasibility of Mxr1-reprogramming strategy in the production of functional recombinant proteins, a secreted single-chain variable fragment (scFv) production strain which contain one copy of P_*AOX1*_-scFv cassette was transformed with linearized Mxr1 reprogrammed plasmid or empty vector. The results of comparison of secreted scFv production between reprogrammed cells KM71Hm/scFv and non-reprogrammed cells KM71H/scFv under methanol induction or glycerol starvation are shown in Fig. 5a. Compared to scFv production in non-reprogrammed cells under methanol induction, scFv expression titre in *P. pastoris* KM71Hm/scFv showed a 98 ± 28% increase under methanol induction and a 269 ± 28% increase under glycerol starvation. These results suggested that the Mxr1-reprogramming strategy show same effects in single copy strain. Notably, western blot analysis revealed that a large amount of scFv remained within *P. pastoris* KM71Hm/scFv under methanol induction, though only a trivial amount was detected under glycerol starvation (Fig. 5b). This accumulation of intracellular scFv in the reprogrammed *P. pastoris* cells suggested that the potential of Mxr1-reprograming was not fully exhibited, as secretion capacity might be the limiting factor in the production of extracellular recombinant proteins. Although further efforts are still required to resolve the observed secretion limitation in methanol-induced Mxr1-reprogrammed *P. pastoris*, recombinant protein production in reprogrammed *P. pastoris* under glycerol starvation appeared to be efficient and applicable.

## Discussion

**Fig. 6:**
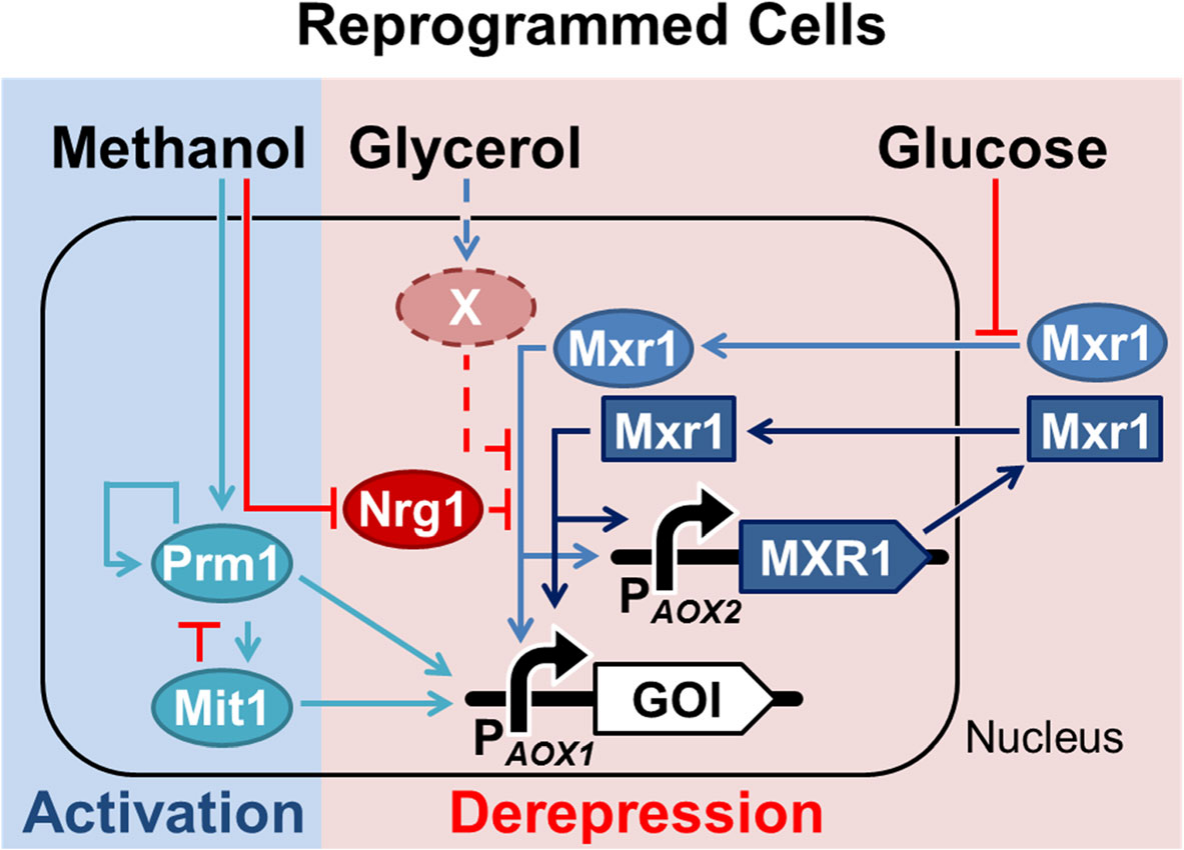
The schematic diagram of transcription regulation in Mxr1-reprogrammed cells. There are two phases, derepression and activation, for P_*AOX1*_ activation in *P. pastoris*. Prm1, Mit1, and Mxr1 are activators (blue). Nrg1 and X are repressors (red). The dashed line and repressor X are speculated based on our results, for which further investigation is needed. In reprogrammed cells, we used P_*AOX2*_ to express extra Mxr1 (dark blue square)

Based on previous studies and the results of this study, we propose a putative mechanism of P_*AOX1*_ transcriptional regulation in reprogrammed *P. pastoris*, as shown in Fig. 6. In this scheme, the exogenous Mxr1 expressed by P_*AOX2*_ establishes a positive auto-regulation circuit in reprogrammed *P. pastoris* cells. When the medium contains glycerol, endogenous Mxr1 enters the nucleus but does not activate P_*AOX1*_ due to the presence of Nrg1 and other repressors [18, 21, 31]. Moreover, the synthetic P_*AOX2*_-regulated circuit is not activated under glycerol repression, and hence reprogrammed *P. pastoris* cells do not exhibit leaky expression or growth defects. When the medium contains methanol, the synthetic Mxr1-reprogrammed circuit is activated in addition to the endogenous regulation machinery [19, 20, 22]. The increased amount of Mxr1 further improves the methanol-induced transcriptional efficiency of P_*AOX1*_ and breaks the limitation of the Mxr1 titration effect [30]. In our study, these is the reason why heterologous gene expression in reprogrammed *P. pastoris* was higher than that in the non-reprogrammed counterpart under methanol induction. In contrast, small amount of residual glycerol did not interfere with the methanol-induced efficiency of P_*AOX1*_ in Mxr1-reprogramed cells, allowing a smooth transition between glycerol and methanol. Besides the plausible explanation that endogenous repression threshold is overcome by the added Mxr1, Zhan et al. indicated that overexpression of Mxr1 represses the expression of glycerol transporter 1 (*gt1*) by binding to the promoter of *gt1* (P_*GT1*_), consequently reducing the content of glycerol in cells and promoting the activation of P_*AOX1*_ [32].

In addition to Mxr1, the role of the repressor Nrg1 was also investigated (Additional file 2: Figure S2). The expression level of Nrg1 was down-regulated in response to methanol. However, there was no significant difference in Nrg1 expression levels between the glycerol group and the no-carbon-source group. It was interesting that the engineered cells could reach high Mxr1 expression level in carbon-free medium without down-regulation of Nrg1. Hence, we speculate the existence of repressor X, which is regulated by glycerol. Under glycerol-starvation conditions, down-regulation of repressor X has a lower inhibitory effect on P_*AOX1*_; however, P_*AOX1*_ is still repressed in non-reprogrammed cells due to weak constitutive expression of endogenous Mxr1. In contrast, down-regulation of repressor X would generate the different strength of repression between glycerol repression and derepression conditions, which explains why P_*AOX1*_ in reprogrammed cells was only activated in glycerol depletion condition but not in glycerol repression condition. Repressor X is likely to be Mig1 or Mig2, as their response to glycerol or methanol is consistent with that of repressor X [3, 22]. Regardless, the detailed expression patterns of Mig1 and Mig2 under glycerol, methanol and carbon depletion require further investigation.

Overexpression or deletion of transcription factors is the simplest way to control P_*AOX1*_ expression; however, these strategies might result in cellular growth defects or break the tight regulation of P_*AOX1*_ in response to repressive carbon sources [23, 32, 33]. Recently, Vogl et al. converted a P_*AOX1*_-based expression strain into a methanol-free production system without breaking the inhibitory effect of glucose through derepressed overexpression of Mxr1 or Mit1 under *CAT1* promoter regulation [23]. Interestingly, a methanol-free production system was also achieved in our study by generating switchable Mxr1 expression via a synthetic positive feedback circuit, even though P_*AOX2*_ is not a naturally derepressed promoter [17, 34]. Compared with the derepressed overexpression of Mxr1, the positive feedback circuit of Mxr1 showed better improvement of productivity under methanol induction and derepression condition. Meanwhile, extra Mxr1 regulated by weak P_*AOX2*_ could prevent the detrimental effects of strong Mxr1 expression [18]. However, both strategies indicate that a synthetic circuit resulting in different levels of transcription factor expression under various conditions is a viable and flexible approach to controlling the characteristics of P_*AOX1*_. Although only the Mut^S^ strain was used in this study, Vogl et al. reported that the Mut phenotype was independent to the methanol regulation machinery [23]. The strategy of transcriptional reprogramming is expected to be effective on both Mut^+^ and Mut^S^ strains.

In addition to the transcriptional process, the secretion pathway is also a bottleneck of recombinant protein production in *P. pastoris* [35, 36]. Previous studies have shown that using a weak promoter could lower the production rate of recombinant proteins, which was favorable for the secretion of proteins with complex folding [14]. Mxr1-reprogrammed *P. pastoris* with the glycerol starvation strategy could achieve the similar goal of slowing down the expression level and get a better productivity of secreted protein. On the other hand, co-expression of target proteins with chaperone protein Kar2 [37], protein disulphide isomerase (PDI) or transcription factors such as Aft1 [38] and Hac1 [39] could enhance the productivity of certain proteins in *P. pastoris* [40]. Hence, the combination of Mxr1 reprogramming with other secretion-related gene circuits is necessary to solving the problem of intracellular target protein accumulation.

## Conclusions

We demonstrated that the recombinant protein production driven by P_*AOX1*_ was greatly enhanced by the synthetic positive feedback circuit of Mxr1 in *P. pastoris* under methanol induction. Breaking the Mxr1 titration effect by added Mxr1 increased the potential of using a high copy-number strategy. In addition, Mxr1 reprogramming reduced interference from the residual repressing carbon source, allowing a smooth transition between different carbon sources. This platform also provided an alternative approach to expressing target genes driven by P_*AOX1*_ under glycerol starvation, thereby eliminating the potential risks of methanol. These Mxr1-reprogrammed cells are expected to have great potential for broader applications.

## Methods

### Plasmids and strains

Standard procedures for the construction of plasmid DNA and transformation of *P. pastoris* were described previously [41]. GFP production cells were first constructed by expressing GFP controlled by the *AOX1* promoter in the Mut^S^ strain *P. pastoris* KM71. Mxr1-reprogrammed cells were then constructed by exogenous expression of Mxr1 controlled by the *AOX2* promoter in a clone carrying nine copies of the GFP expression cassette. Empty vector control cells were constructed to restore the histidine deficiency of the GFP production clone. In both the empty vector control and Mxr1-reprogrammed cells, clones with only one copy of the P_*AOX2*_-regulated cassette were selected for further analysis and named KM71/GFP and KM71m/GFP, respectively. Single-chain variable fragment (scFv) production cells were obtained by transforming pPICZαA-scFv into *P. pastoris* KM71H. The pAOX2KH and pAOX2KH-Mxr1p plasmids were transformed separately into the clone carrying one copy of the scFv expression cassette to generate the empty vector control and Mxr1-reprogrammed cells, which were named KM71H/scFv and KM71Hm/scFv, respectively. The strains used in this study and their characteristics are listed in Table 1. The primers used for plasmids construction and sequencing are provided in Additional file 3: Table S1.

**Table 1:** Strains used in this study

Strain	Characteristics	Source
*E. coli* EPI300	Gene cloning host	Epicentre Technologies Corp, USA
*P. pastoris* KM71	Gene expression host with histidine deficiency	Invitrogen, USA
*P. pastoris* KM71H	Gene expression host	Invitrogen, USA
*P. pastoris* KM71/GFP	GFP expressed in *P. pastoris* KM71 and histidine deficiency was restored by the empty pAOX2 vector	In this study
*P. pastoris* KM71m/GFP	GFP expressed in *P. pastoris* KM71m containing the synthetic Mxr1 circuit	In this study
*P. pastoris* KM71H/scFv	scFv expressed in *P. pastoris* KM71H	In this study
*P. pastoris* KM71Hm/scFv	scFv expressed in *P. pastoris* KM71Hm containing the synthetic Mxr1 circuit	In this study

### Media and culture conditions

The media used in this study are listed in Additional file 4: Table S2. The protein expression procedure for *P. pastoris* was conducted according to the manufacturer’s instructions (Invitrogen, Carlsbad, California, USA). Cells were cultured in 3 mL YPDZ for 20 h as seed culture and inoculated into 100 mL BMGY medium to an optical density at 600 nm (OD_600_) of 0.15. The cells were grown at 30 °C, 250 rpm for 24 h and harvested by centrifugation at 3000×*g* for 10 min to remove residual repressive carbon sources in the supernatant. The pellet was resuspended in 20 mL of a different carbon source medium, BMMY, BMGY, or BMNY. BMGY was a fresh medium with a different concentration of glycerol. BMMY was a fresh medium with 0.5% methanol added every 24 h to induce protein expression. BMNY was a fresh medium without a carbon source.

### Protein expression analysis

Quantification of GFP expression was monitored using a SpectraMax M2e Microplate Reader (Molecular Device, Sunnyvale, California, USA). The excitation wavelength was 488 nm, with an emission wavelength of 509 nm. The GFP expression level was normalized to the cell density, which was monitored by optical density at 600 nm. Extracellular expression of scFv was analysed by sodium dodecyl sulphate polyacrylamide gel electrophoresis (SDS-PAGE), followed by Coomassie Brilliant Blue G-250 staining. The relative scFv titre was determined using UVP image analysis software (Analytik Jena AG, Jena, Germany). For intracellular protein analysis, cell pellets were harvested by centrifugation and washed once with the same volume of lysis buffer (50 mM monosodium phosphate pH 7.4, 1 mM ethylenediaminetetraacetic acid (EDTA), 5% glycerol, 1 mM phenylmethylsulfonyl fluoride (PMSF)). The cell pellets were resuspended in breaking buffer and ground in liquid nitrogen. Total intercellular proteins were transferred to a polyvinylidene fluoride (PVDF) membrane (PerkinElmer, Waltham, Massachusetts, USA) after electrophoresis and detected by specific antibodies. The primary antibody was a rabbit anti-his polyclonal antibody (Bioman, New Taipei City, Taiwan), and a horseradish peroxidase (HRP)-conjugated antibody (PerkinElmer) was used as the secondary antibody. Both antibodies were diluted 5000× with gelatine-NET (0.15 M NaCl, 5 mM EDTA, 0.05% Tween in 50 mM Tris-HCl, pH 8.0) before use. Colorimetric detection was performed using an enhanced chemiluminescence substrate (PerkinElmer). The total intracellular protein content was assessed using a rabbit anti-GAPDH polyclonal antibody (GeneTex, Irvine, California, USA).

### RNA expression level analysis

The mRNA expression levels of transcription factor genes and GFP were verified by real-time PCR. Total RNA derived from cells was extracted using a NautiaZ Bacteria/Fungi RNA Mini Kit (Nautia Gene, Taipei, Taiwan) in accordance with the manufacturer’s procedure. Reverse transcription was performed using ARROW-Script Reverse transcriptase III with Radom hexamers (ARROWTEC, Taipei, Taiwan), and the products were used for subsequent real-time PCR performed with the StepOne™ System (Applied Biosystems, Foster, California, USA) using 2xIQ^2^ SYBR Green FAST qPCR System Master Mix-HIGH ROX (Bio-Genesis Technologies, Taipei, Taiwan). The primers used for real-time PCR are listed in Additional file 5: Table S3. After the cycle threshold values (CT) were determined, relative fold differences were calculated using the 2^−ΔΔCT^ method with 18S rRNA as the endogenous reference gene.

### AOX activity assay

Cells were cultured in 3 mL YPDZ for 16 h as seed culture and inoculated into 3 mL BMDY, BMGY, BMMY, BMNY, or BMGMY medium to an optical density of 1at 600 nm (OD_600_). The cells were grown at 30 °C, 250 rpm for 10 h, and a total of 5 × 10^7^ cells was harvested by centrifugation. The cell pellets were resuspended in AOX activity reagent and incubated at 30 °C for 30 min [42].

## Additional files

Additional file 1: **Figure S1.** The delta CT values of GFP (left) and *MXR1* (right) expression. The mRNA was extracted from the cells cultured in different carbon sources for 3 h. The mRNA levels were normalized to 18S rRNA in each sample and represented by delta C value. The error bars represented the standard deviation of three biological replicates. (DOCX 46 kb)

Additional file 2: **Figure S2.** The mRNA expression level of *NRG1*. The mRNA was extracted from the cells cultured in different carbon sources for 3 h. The mRNA levels were normalized to 18S rRNA in each sample. The relative expression level for each gene was normalized to the control grown in the carbon-free condition. The error bars represented the standard deviation of three biological replicates. The two-way ANOVA and Turkey test were used to determine the statistical significance. The groups with different alphabet were significantly different. (DOCX 1483 kb)

Additional file 3: **Table S1.** The primers used for plasmids construction and sequencing. (DOCX 12 kb)

Additional file 4: **Table S2.** The media used in this study. (DOCX 12 kb)

Additional file 5: **Table S3.** The primers used for real-time PCR. (DOCX 12 kb)

Additional file 6: Excel file of raw data generated or analyzed during this study. (XLSX 554 kb)

## Abbreviations

AOX: Alcohol oxidase
BMDY: Buffered dextrose-complex medium
BMGY: Buffered glycerol-complex medium
BMMY: Buffered methanol-complex medium
BMNY: Buffered complex medium without a carbon source
GFP: green Fluorescent protein
KM71m: Mxr1-reprogrammed KM71 strains
Mxr1: Methanol expression regulator 1 P_*AOX1*_ Alcohol oxidase 1 promoter
scFv: Single-chain variable fragment
YPDZ: Yeast extract-peptone-dextrose with zeocin
