# Supplementary material for: Enhancing the efficiency of the Pichia pastoris AOX1 promoter via the synthetic positive feedback circuit of transcription factor Mxr1
Source: BMC Biotechnol. 2018 Dec 27;18:81. doi: 10.1186/s12896-018-0492-4 (PMC6307218; doi:10.1186/s12896-018-0492-4)
Supplement: Supplementary file 1 — Figure S1. The delta CT values of GFP (left) and MXR1 (right) expression. The mRNA was extracted from the cells cultured in different carbon sources for 3 h. The mRNA levels were normalized to 18S rRNA in each sample and represented by delta C value. The error bars represented the standard deviation of three biological replicates. (DOCX 46 kb) [file 12896_2018_492_MOESM1_ESM.docx]

**
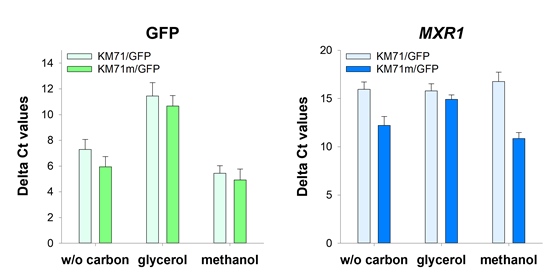
**

**Figure S1. The delta CT value of GFP (left) and *MXR1* (right) expression.**

The mRNA was extracted from the cells cultured in different carbon sources for 3 hours. The mRNA levels were normalized to 18S rRNA in each sample and represented by delta C value. The error bars represented the standard deviation of three biological replicates.
