# Supplementary material for: Enhancing the efficiency of the Pichia pastoris AOX1 promoter via the synthetic positive feedback circuit of transcription factor Mxr1
Source: BMC Biotechnol. 2018 Dec 27;18:81. doi: 10.1186/s12896-018-0492-4 (PMC6307218; doi:10.1186/s12896-018-0492-4)
Supplement: Supplementary file 2 — Figure S2. The mRNA expression level of NRG1. The mRNA was extracted from the cells cultured in different carbon sources for 3 h. The mRNA levels were normalized to 18S rRNA in each sample. The relative expression level for each gene was normalized to the control grown in the carbon-free condition. The error bars represented the standard deviation of three biological replicates. The two-way ANOVA and Turkey test were used to determine the statistical significance. The groups with different alphabet were significantly different. (DOCX 1483 kb) [file 12896_2018_492_MOESM2_ESM.docx]

**
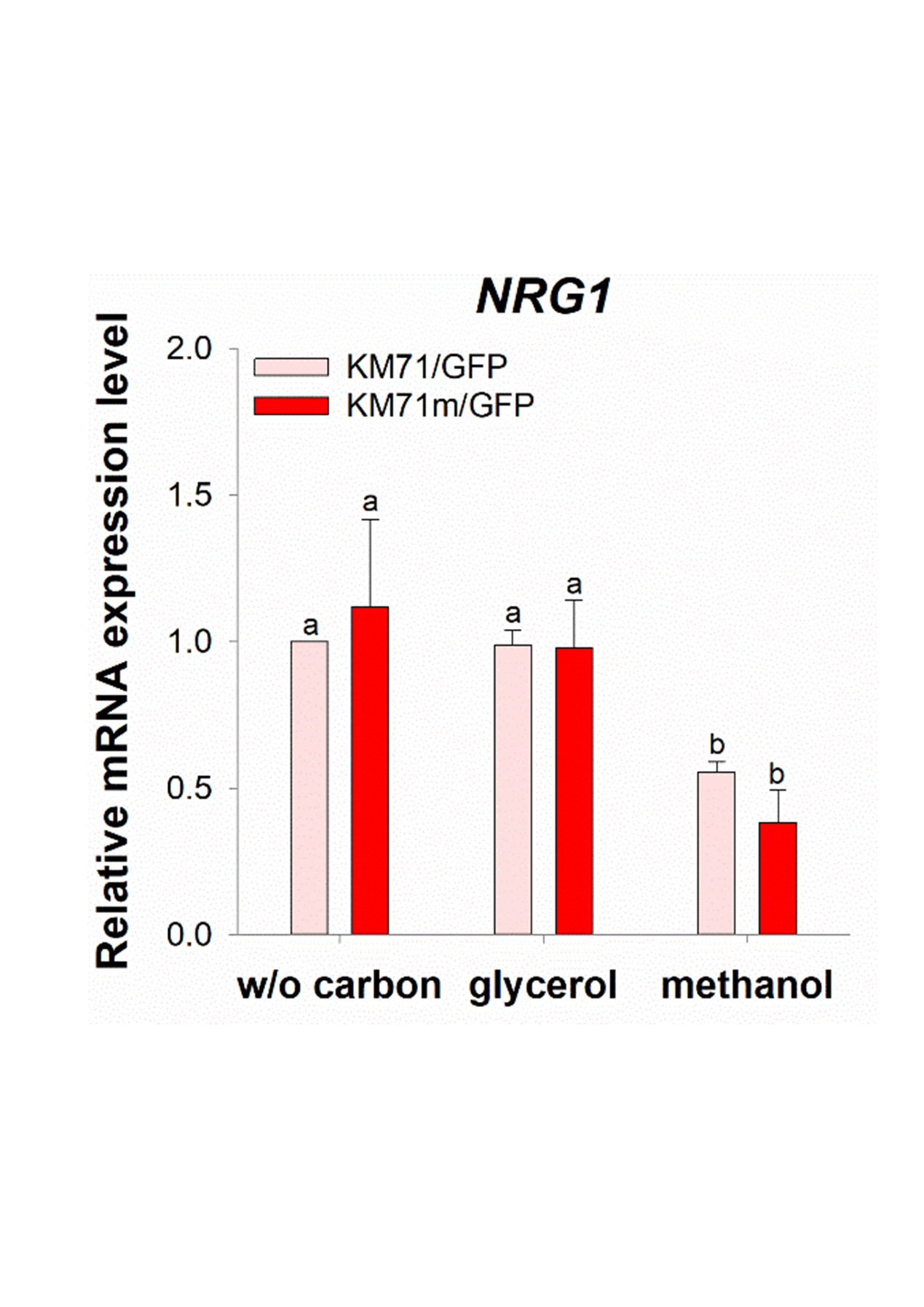
Figure S2. The mRNA expression level of *NRG1*.**

The mRNA was extracted from the cells cultured in different carbon sources for 3 hours. The mRNA levels were normalized to 18S rRNA in each sample. The relative expression level for each gene was normalized to the control grown in the carbon-free condition. The error bars represented the standard deviation of three biological replicates. The two-way ANOVA and Turkey test were used to determine the statistical significance. The groups with different alphabet were significantly different.
