# Supplementary material for: Enhancing the efficiency of the Pichia pastoris AOX1 promoter via the synthetic positive feedback circuit of transcription factor Mxr1
Source: BMC Biotechnol. 2018 Dec 27;18:81. doi: 10.1186/s12896-018-0492-4 (PMC6307218; doi:10.1186/s12896-018-0492-4)
Supplement: Supplementary file 3 — Table S1. The primers used for plasmids construction and sequencing. (DOCX 12 kb) [file 12896_2018_492_MOESM3_ESM.docx]

Table S1. The primers used for plasmids construction and sequencing.

| Primer | Sequence (5' end to 3' end) |
| --- | --- |
| F-*Pml*I-GFP | CACGTGATGGTGAGCAAGGGC |
| R-GFP-*Sal*I | accgtcgacttacttgtacagctcg |
| F-*Avr*II-6xHis | ccctaggcatcatcatcatcatcattgatcaa gaggatgtc |
| R-partialHIS4-*Xba*I | catctagatgctcaccgcaatgctg |
| F-*Aat*II-AOX2 | gcggacgtctttttttcagacc |
| R-AOX2-*Bam*HI | gcgggatcctttttctcagttg |
| F-*Bam*HI-Mxr1 | gcgggatccatgagcaatctacc |
| R-SalImutant-Mxr1 | CCCAGTTCTTAGTGGACTCATTCTCATC |
| F-SalImutant-Mxr1 | gatgagaatgagtccactaagaactggg |
| R-MXR1P-*Avr*II | gcgcctagggacaccaccatcta |
| F-*Eco*RI-scFv | GAGCCGAATTCCAAGTTCAATTGAAG |
| R-scFv-*Sal*I | GCCGTCGACTTTCAACTCCAAC |
