# Supplementary material for: Enhancing the efficiency of the Pichia pastoris AOX1 promoter via the synthetic positive feedback circuit of transcription factor Mxr1
Source: BMC Biotechnol. 2018 Dec 27;18:81. doi: 10.1186/s12896-018-0492-4 (PMC6307218; doi:10.1186/s12896-018-0492-4)
Supplement: Supplementary file 4 — Table S2. The media used in this study. (DOCX 12 kb) [file 12896_2018_492_MOESM4_ESM.docx]

Table S2. The media used in this study.

| Meida | Composition |
| --- | --- |
| BMDY | 2% dextrose, 1% yeast extract, 2% peptone, 100 mM potassium phosphate (pH 6.0), 1.34% YNB, 4 × 10^-5^% biotin |
| BMGY | 1%, 2%, or 4% glycerol, 1, 1% yeast extract, 2% peptone, 100 mM potassium phosphate (pH 6.0), 1.34% YNB, 4 × 10^-5^% biotin |
| BMMY | 0.5% methanol, 1% yeast extract, 2% peptone, 100 mM potassium phosphate (pH 6.0), 1.34% YNB, 4 × 10^-5^% biotin |
| BMGMY | 0.1% glycerol, 0.1% methanol, 1% yeast extract, 2% peptone, 100 mM potassium phosphate (pH 6.0), 1.34% YNB, 4 × 10^-5^% biotin |
| BMNY | 1% yeast extract, 2% peptone, 100 mM potassium phosphate (pH 6.0), 1.34% YNB, 4 × 10^-5^% biotin |
