# Supplementary material for: Enhancing the efficiency of the Pichia pastoris AOX1 promoter via the synthetic positive feedback circuit of transcription factor Mxr1
Source: BMC Biotechnol. 2018 Dec 27;18:81. doi: 10.1186/s12896-018-0492-4 (PMC6307218; doi:10.1186/s12896-018-0492-4)
Supplement: Supplementary file 5 — Table S3. The primers used for real-time PCR. (DOCX 12 kb) [file 12896_2018_492_MOESM5_ESM.docx]

Table S3. The primers used for real-time PCR.

| Primer | Sequence (5' end to 3' end) |
| --- | --- |
| Copy-MET2-qF | CGTTCTCGCAACTCTTTCGAAGAA |
| Copy-MET2-qR | CAATGGCATCAGTTATGACGGAAG |
| Copy-AOX1p-qF | TTCTCACACATAAGTGCCAAACG |
| Copy-AOX1p-qR | AAAAGTGGGTGTTGAGGAGAAGAG |
| Copy-AOX2p-qF | CACCCAGCCCTCTTCATCAA |
| Copy-AOX2p-qR | TCGCCCTTGGATGGAAAA |
| 18s rRNA-qF | GAGGATTGACAGGATGAGAGC |
| 18s rRNA-qR | CAAGGTCTCGTTCGTTATCGC |
| GFP-qF | GGCACAAGCTGGAGTACAACT |
| GFP-qR | ATGTTGTGGCGGATCTTGAAG |
| MXR1-qF | CTGATGCTATGAATGCCAAGGA |
| MXR1-qR | CTGAATCGTTATTACGACCGGAAT |
| PRM1-qF | TGGCGGCAAGAAGAACGTATA |
| PRM1-qR | TGGTTGCTGTGCCTTTGCTA |
| MIT1-qF | GTGGCACGCTGGAAAGCTAAT |
| MIT1-qR | ATAGGCCAGCCTGTTGCACA |
| NRG1-qF | CCCAACACAGCCATCAGAAA |
| NRG1-qR | GTGAATACGTGTGTGACGAGC |
